# Gothelf’s Haplotype of COMT in Parkinson’s Disease: A Case–Control Study

**DOI:** 10.3390/biomedicines14020262

**Published:** 2026-01-23

**Authors:** Zdenko Červenák, Ján Somorčík, Žaneta Zajacová, Andrea Gažová, Igor Straka, Zuzana André, Michal Minár, Ján Kyselovič

**Affiliations:** 1 5th Department of Internal Medicine, Faculty of Medicine, Comenius University, Spitalska 24, 813 72 Bratislava, Slovakia; zdenko.cervenak@fmed.uniba.sk (Z.Č.); kyselovic@uniba.sk (J.K.); 2 Department of Applied Mathematics and Statistics, Faculty of Mathematics, Physics and Informatics, Comenius University, Mlynská dolina F1, 842 48 Bratislava, Slovakia; jan.somorcik@fmph.uniba.sk; 3 Department of Pharmacology and Toxicology, Faculty of Pharmacy, Comenius University, Odbojárov 10, 832 32 Bratislava, Slovakia; zajacova76@uniba.sk; 4 Institute of Pharmacology and Clinical Pharmacology, Faculty of Medicine, Comenius University, Spitalska 24, 813 72 Bratislava, Slovakia; 5 2nd Department of Neurology, Faculty of Medicine, Comenius University, Spitalska 24, 813 72 Bratislava, Slovakia; straka0105@gmail.com (I.S.); zuzana.andre@fmed.uniba.sk (Z.A.); michal.minar@fmed.uniba.sk (M.M.)

**Keywords:** COMT, Parkinson’s disease, haplotype, L-dopa, SNPs

## Abstract

**Background:** Catechol-O-methyltransferase (COMT) catalyzes catecholamine O-methylation and contributes to dopamine turnover, potentially influencing levodopa requirements in Parkinson’s disease (PD). We evaluated whether the Gothelf COMT haplotype—and its constituent variants rs2075507, rs4680 (Val158Met), and rs165599—differ in frequency between PD cases and controls. We then tested associations between these variants and clinical phenotypes, with a prespecified focus on levodopa equivalent daily dose (LEDD). Finally, we examined whether haplotype structure and allele-specific context (e.g., background-dependent effects) help explain observed genotype–phenotype relationships in the PD cohort. **Aim:** Analysis of the rs2075507, rs4680 and rs165599 at individual and haplotype level between control and diseased groups. Furthermore, analysis of association of individual SNPs or haplotype level with clinical outcomes. **Subjects and methods:** Fifty-five individuals with Parkinson’s disease (PD) and fifty-three neurologically healthy controls were enrolled at a single center. Genomic DNA was isolated from peripheral blood, and three COMT variants—rs2075507 (promoter), rs4680/Val158Met (coding), and rs165599 (3′UTR)—were genotyped by Sanger sequencing. Allele, genotype, and tri-marker haplotype frequencies were estimated, and case–control differences were evaluated. Within the PD cohort, associations with clinical outcomes—primarily levodopa equivalent daily dose (LEDD)—were analyzed using multivariable linear models. Statistical tests were two-sided, with multiplicity control as specified in the corresponding tables. **Results:** The rs2075507 polymorphism showed a robust additive association with LEDD; each A allele predicted higher dose (LEDD ≈ +1331 mg/day, *p* = 0.001) after adjusting for age and sex. The tri-haplotype test did not show significant association with LEDD. Nevertheless, rs2075507 SNP strongly marked downstream backgrounds: in AA carriers, rs4680–rs165599 haplotypes were enriched for Val (G) and rs165599-G; in GG carriers, for rs165599-A with mixed Val/Met; and GA was A-loaded at both loci. Exact tests confirmed that AA and GG differed in rs4680–rs165599 composition, whereas GA vs. GG was not significant. **Conclusions:** The promoter variation at rs2075507 may represent the genetic contributor to levodopa dose requirements when modeled with SNP–SNP interactions, with its effect is modified mostly by rs165599 polymorphism. Tri-haplotypes do not independently predict LEDD. The rs4680 (coding) and rs165599 (3′UTR) context appears to fine-tune rather than determine dosing needs, mainly via interaction with rs2075507 SNP.

## 1. Introduction

Parkinson’s disease (PD) is a progressive neurodegenerative disorder characterized by a combination of motor symptoms, including bradykinesia, resting tremor, and muscular rigidity, and non-motor symptoms such as cognitive decline, sleep disturbances, and neuropsychiatric alterations. Although the precise etiology of PD remains elusive, it is widely accepted that the disease results from a multifactorial interplay of genetic predispositions and environmental exposures, culminating in the selective degeneration of dopaminergic neurons in the substantia nigra and consequent central dopamine deficiency [1].

In the central nervous system, dopamine is predominantly degraded by monoamine oxidase B (MAOB) and catechol-O-methyltransferase (COMT) [2]. COMT catalyzes the O-methylation of catecholamines, thereby inactivating dopamine and metabolizing potentially neurotoxic catechols and hydroxylated metabolites [3]. Reduced peripheral COMT activity diminishes L-DOPA (levodopa) degradation, thereby enhancing its central bioavailability and therapeutic efficacy [4]. Variations in COMT enzymatic activity—modulated by genetic polymorphisms—may therefore contribute to inter-individual differences in response to dopaminergic treatments, including levodopa and COMT inhibitors such as entacapone, and may potentially influence disease trajectory [5,6,7].

Over the past two decades, numerous single nucleotide polymorphisms (SNPs) located within both coding (exonic) and noncoding regions of the COMT gene have been investigated, identifying a range of genetic variants associated with differential enzymatic activity. The majority of studies have focused on the rs4680 polymorphism, a non-synonymous G-to-A substitution that results in a valine-to-methionine (Val158Met) amino acid change in exon 4. This substitution has been shown to reduce COMT enzymatic activity by approximately 40% in human dorsolateral prefrontal cortex tissue and lymphoblastoid cell cultures [8]. Additional well-characterized variants, such as rs4818 and rs4633, significantly influence the transcriptional and translational regulation of COMT [9,10,11]. Moreover, several studies have highlighted the role of two further SNPs—rs2075507 and rs165599—both located in noncoding regions of the gene [8,12,13]. The rs2075507 variant, positioned near the P2 promoter, modulates the expression of the membrane-bound isoform MB-COMT in the brain, whereas rs165599, located in the 3′ untranslated region (3′UTR) of a brain-specific transcript, may alter post-transcriptional regulation through interaction with microRNAs [8,14,15,16]. Both polymorphisms have been extensively studied in the context of schizophrenia [13,17,18,19], yet their role in PD susceptibility and progression remains poorly characterized.

Several studies have demonstrated that the combination of single nucleotide polymorphisms (SNPs) into haplotypes can influence COMT activity in ways that differ from the effects of individual variants alone. This was first highlighted by Nackley, who showed that four common SNPs in the COMT gene (rs6269, rs4633, rs4818, rs4680) form functionally distinct haplotypes associated with differential COMT enzymatic activity and pain sensitivity. They identified three major haplotypes: A_C_C_G, linked to the lowest COMT activity and highest pain sensitivity; A_T_C_A, associated with intermediate activity; and G_C_G_G, associated with the highest activity. These combinations, now widely known as “Nackley’s haplotypes”, demonstrated that haplotype structure rather than single polymorphisms may provide a more accurate predictor of COMT function [9,20]. It has been reported that the rs4633-rs4680 haplotype is associated with several clinical parameters in a Chinese patient with PD [21] and full haplotypes were tested for potential relation with clinical outcomes of PD with contradictory results [21,22,23]. Further work by Gothelf extended these findings to neuropsychiatric and cognitive domains. In their 2005 longitudinal study of individuals with 22q11.2 deletion syndrome, they reported that COMT genotype predicts cognitive decline and psychosis risk, thereby linking COMT variation to clinically relevant outcomes [24]. In a later study, Gothelf et al. [25] investigated the biological effects of COMT haplotypes in the same syndrome and demonstrated that haplotype configurations significantly influence COMT mRNA expression, enzymatic activity, and psychosis susceptibility. Together, these studies highlight the importance of considering COMT haplotypes—rather than individual SNPs—in understanding variability in enzyme function and its consequences for pain perception, cognition, and psychiatric risk.

The objective of this study was to investigate three COMT polymorphisms—rs2075507 (promoter), rs4680/Val158Met (coding), and rs165599 (3′UTR)—which together define the Gothelf haplotype [25] in a case–control cohort. We evaluated allele, genotype, and tri-marker haplotype frequencies in Parkinson’s disease (PD) versus controls, and examined associations with selected clinical phenotypes in PD, with a prespecified focus on levodopa equivalent daily dose (LEDD). Variant–LEDD associations were assessed both for individual SNPs and for their haplotype combinations.

## 2. Materials and Methods

### 2.1. Subjects

In this study, we included 55 patients with Parkinson’s disease from the Second Department of Neurology, Faculty of Medicine, Comenius University and University Hospital in Bratislava. PD was diagnosed according to the UK-PD Society Brain Bank Criteria [26] and the MDS clinical diagnostic criteria for PD [27]. Among the patients, 5 subjects were diagnosed with early onset of PD (EOPD, onset before 50 years of age) and 50 with late onset (LOPD, onset after 50 years of age); 8/55 of PD patients had diabetes mellitus (all type 2) and 22/55 had arterial hypertension. The progression of the disease was evaluated using the severity of the Hoehn and Yahr scale, the motor symptoms using the Unified Parkinson’s Disease Rating Scale (UPDRS), part III (in ON phase) and the therapy using UPDRS, part IV. Other clinical and demographic characteristics included age of disease onset, disease duration, and LEDD (levodopa equivalent of daily dose, calculated according to [28]).

Control samples were obtained from 53 randomly selected individuals hospitalized in the same hospital without any diagnosed CNS disease or symptoms. The study was approved by regulatory authorities and the local ethics committee (Ethics Committee of the University Hospital in Bratislava; Decision numb.: 13/2021), and all participants provided their written informed consent.

### 2.2. Genetic Analysis

Genomic DNA was extracted from peripheral blood samples collected from each individual using the QIAamp DNA Blood Mini Kit (Qiagen GmbH, Hilden, Germany) according to the manufacturer’s protocol. Genotyping was performed on four PCR amplicons (primer sequences, annealing temperatures, and amplicon sizes for all amplicons used in COMT genetic polymorphism analysis are presented in Supplementary Materials, Table S1, Primers). Purified PCR products were sequenced using BigDye Termintor v3.1 chemistry on SeqStudio Genetic Analyzer (Thermo Fisher Scientific, Waltham, MA, USA).

### 2.3. Statistical Analysis

Associations between categorical variables were assessed using Fisher’s exact test, while continuous variables were analyzed using the nonparametric Mann–Whitney U test. Hardy–Weinberg equilibrium (HWE) concordance, as well as comparisons of genotype and allele frequencies between PD patients and controls, haplotype inference from genotype data, and intergroup haplotype frequency distribution comparisons were performed using Arlequin software ver. 3.5 [29], employing appropriate modules tailored to each analysis type. The linkage disequilibrium (LD) between all three SNPs was estimated using Haploview software version 4.2 “www.broad.mit.edu/mpg/haploview (accessed on 30 June 2024)”.

Associations between three COMT polymorphisms and clinical parameters were examined using classical linear regression based on the least square method, incorporating both categorical and continuous predictors (age and sex as covariate). The impact of individual SNPs on the distribution of Hoehn and Yahr stages was evaluated using Pearson’s chi-squared homogeneity test with simulated *p*-values. Statistical significance was set at *p* ≤ 0.05, with Holm correction for multiple testing applied. All statistical analyses were conducted using GraphPad Prism version 10.4.1 and XLSTAT (Addinsoft, 2025).

## 3. Results

Table 1 summarizes the basic demographic and clinical characteristics of the study participants. No significant differences in age were observed between the Parkinson’s disease and control groups, nor between males and females within either cohort. The control group included a significantly higher proportion of female participants compared to the PD group. Within the PD cohort, no significant sex-based differences were found for disease duration, age at onset, daily levodopa equivalent dose, UPDRS Part III and IV scores, or Hoehn and Yahr stage.

The distribution of genotypes and alleles for three COMT gene polymorphisms (rs2075507, rs4680, and rs165599) in both the control group and PD patients is summarized in Table 2.

Minor deviation from HWE was observed in the control group for rs4680; however, it became nonsignificant after correction for multiple testing. A comparison of genotype and allele frequencies between healthy controls and PD patients revealed no significant difference in genotype distribution for any polymorphism. The LD mapping (Supplementary Materials, Table S2, Linkage disequilibrium) showed that LD of rs4680-rs165599 is moderate and partially correlated but not interchangeable. The rs2075507-rs4680/rs165599 LD is weak-to-moderate (lower r^2^ than the pair above) indicating that rs2075507 SNP carries substantial independent information relative to the coding (rs4680) and 3′UTR (rs165599) sites.

Furthermore, analysis of the haplotype composed of rs2075507, rs4680, and rs165599 SNPs has shown no significant differences between patients and controls (Supplementary Materials, Table S3, Haplotype frequency comparison).

The associations between selected clinical characteristics (disease onset, duration, LEDD, Hoehn and Yahr and UPDRS III and IV score) and all three SNPs were also evaluated. No significant relationships were identified between individual polymorphisms and clinical parameters except for the relation of rs2075507 SNP to LEDD. Our analysis has revealed significant difference for the rs2075507 A allele under additive model with interactions (Supplementary Materials, Table S4, Multivariable analysis 1, *p* = 0.007; dominant and recessive models non-significant).

We found that patients carrying the rs2075507 AA genotype exhibited higher LEDD requirements compared to those with the GA or GG genotypes (AA: 1760.58 ± 679.40 mg/day; GA: 1204.77 ± 603.77 mg/day; GG: 1357.61 ± 522.94 mg/day; values expressed as mean ± standard deviation, significant for AA vs. GA comparison, MW test, *p* = 0.025, Supplementary Materials, Table S5, Analysis of demographic characteristics and clinical outcomes). The haplotype analysis has shown no significant association of the individual haplotypes with LEDD (Supplementary Materials, Table S6, Multivariable analysis 2) with age showing a strong, consistent positive effect (~+17–18 mg/day per year). Interestingly, nearly all haplotypes with G allele at rs2075507 have exerted the lower even not significant LEDD doses when compared with A allele at the same position (Supplementary Materials, Table S6, Multivariable analysis 2).

In the additive model with pairwise interactions, rs2075507 A shows a large per-allele increase in LEDD in the rs165599 = GG background, with attenuation per rs165599 A allele; thus, the rs2075507 effect may depend on haplotype context, mainly rs165599 SNP (Supplementary Materials, Table S4, Multivariable analysis 1). Therefore, we examined how the rs4680 and rs165599 polymorphisms (and their haplotypes) distribute across the three rs2075507 genotypes. First, allele-level comparisons indicated that rs2075507 AA homozygotes differ from both GG homozygotes and GA heterozygotes in the allele frequencies of rs4680 and rs165599 (Table 3). Consistently, the two-locus rs4680–rs165599 haplotype analysis showed significant differences between AA and each of the other two rs2075507 groups, but no difference between GA and GG (Table 4; AA vs. GA *p* = 0.002; AA vs. GG *p* = 0; GA vs. GG *p* = 0.099; Holm-corrected). In terms of dosing, higher LEDD tended to be associated with the G–G di-haplotype at rs4680–rs165599 (Supplementary Materials, Table S7, Multivariable analysis 3), although this trend did not reach statistical significance. We also observed an LEDD difference between AGA and AGG three-locus haplotypes, suggesting that the rs165599 G allele may modulate COMT activity (Supplementary Materials, Table S6, Multivariable analysis 2). A similar trend appeared across haplotypes carrying G at rs2075507. Notably, GG and GA genotypes at rs2075507 showed comparable mean LEDD, raising the possibility that G allele at rs2075507 (or a polymorphism in linkage disequilibrium with it) reduces COMT activity relative to A, independent of zygosity at this promoter site.

## 4. Discussion

Most case–control studies of COMT and Parkinson’s disease have centered on rs4680 (Val158Met). Findings are heterogeneous: several European cohorts (Greek, Polish, Finnish) report no association [20,30,31], whereas some Asian studies suggest increased risk [32,33]. In our cohort, genotype and allele frequencies at rs4680 did not differ between PD and controls. Likewise, the promoter variant rs2075507 (P2 region) and rs165599 showed no case–control differences, consistent with most European data. These results contrast with population-specific signals reported elsewhere (e.g., Ashkenazi Jewish cohorts for rs165599; [13]) and likely reflect ethnic and genetic heterogeneity, LD architecture, and phenotype differences rather than a uniform effect of common COMT alleles on PD risk in Europeans.

When we assessed Gothelf’s haplotype framework, we observed no case–control differences in haplotype frequencies. Similar negative case–control findings were reported in a Turkish schizophrenia cohort [16] and for the Nackley 4-SNP haplotypes (rs6269–rs4633–rs4818–rs4680) in Polish and Chinese PD cohorts [21,22].

For clinical correlations, we detected a single robust association: rs2075507 and LEDD. Carriers of the AA genotype required significantly higher LEDD than GA or GG. Prior work within Gothelf’s framework reported that haplotypes carrying A at rs2075507 are associated with higher COMT enzymatic activity compared with haplotypes carrying G, and that activity also varies with rs4680 (G/Val > A/Met) and rs165599 (A > G) compositions [25]. To compare our dosing result to Gothelf’s activity readouts—recognizing that phenotypes differ—we used the working assumption that higher COMT activity → higher LEDD (faster dopamine catabolism increases dose needs). Under this assumption, our finding that rs2075507 = AA (A-enriched backgrounds) shows higher LEDD is directionally consistent with Gothelf’s report that A at rs2075507 marks higher activity backgrounds. At the haplotype level, our highest LEDD mean appeared with the AGG (rs2075507–rs4680–rs165599) haplotype, which does not match Gothelf’s ranking (AGG was fourth in activity there). Several technical factors can explain this divergence: (i) we analyze 3-marker haplotypes rather than the full functional context; (ii) LEDD is a clinic-level endpoint (age, duration, weight, adjuncts, prescriber effects) and introduces variance that can reorder means; (iii) LD differences in our cohort alter how 3-marker combinations mark functional backgrounds; and (iv) some haplotypes are rare, inflating SEs and destabilizing ranks.

Haplotype frequency patterns across rs2075507 strata have shown that in rs2075507 = AA, the G–G (rs4680–rs165599) di-haplotype accounted for ~62% of haplotypes, but only ~7% in GG and ~30% in GA (Table 4), and AGG showed higher LEDD consumption. This superficially contrasts with reports that G–G associates with the lower COMT expression/activity in other tissues [12,25]. Gothelf also noted that rs165599 effects are contingent on the rs4680 background (stronger on G/Val chromosomes, negligible on A/Met), and in our cohort we observed a non-significant trend to lower LEDD in rs165599 = G carriers regardless of rs4680—consistent with a fine-tuning rather than a primary driver.

LD structure helps further reconcile these observations. In the PD cohort, pairwise LD was moderate (r^2^ ≈ 0.28–0.50) among the three COMT SNPs, with the strongest correlation between rs4680 and rs165599 and the weakest between rs2075507 and rs165599 (Supplementary Material, Table S2, Linkage disequilibrium analysis). This pattern suggests that the observed frequency distribution of rs4680–rs165599 haplotypes primarily reflects their underlying linkage disequilibrium, rather than independent haplotypic effects. However, the rs2075507–rs165599 interaction indicates context-dependent effects not attributable solely to allelic correlation from linkage disequilibrium, consistent with haplotype-dependent modulation of the rs2075507 promoter effect. Moreover, the high homozygosity observed at rs2075507 (AA and GG; is compatible with extended haplotype structure in this region, supporting the view that multi-locus background, rather than single polymorphisms in isolation, contributes to variation in LEDD requirements in this cohort.

In our cohort, rs2075507 reveals a context-dependent (allele-specific) influence of rs165599 on dose requirements. Because rs165599 lies in the 3′UTR, an miRNA-mediated mechanism is plausible: published data indicate that miR-138-5p preferentially represses the rs165599-G allele, whereas miR-22-3p binds more strongly when the A allele is present [15,16]. Together with the known functional coding variant rs4680 (Val/Met), these observations suggest that post-transcriptional control at rs165599 may fine-tune, but does not override, the enzymatic backdrop set by rs4680 and the rs2075507 promoter. Practically, the rs2075507 effect is large and positive in the rs165599 = GG background and attenuates with A copies at rs165599, aligning higher-COMT-activity backgrounds with higher LEDD, and lower-activity backgrounds with lower LEDD.

Taken together, our results fit a working model where rs2075507 establishes a promoter-driven baseline of MB-COMT, rs4680 tunes catalytic activity, and rs165599 adds allele-specific miRNA-mediated fine-tuning; together they shape LEDD, with rs2075507 providing the primary directionality and rs4680/rs165599 modulating its magnitude within rs2075507 backgrounds.

## 5. Conclusions

In summary, promoter variation at rs2075507 may represent the genetic contributor to levodopa dose requirements when modeled with SNP interactions, with its effect modified mostly by rs165599 polymorphism. Tri-haplotypes do not independently predict LEDD. The rs4680 (coding) and rs165599 (3′UTR) context appears to fine-tune rather than determine dosing needs, mainly via interaction with rs2075507 SNP. Across our SNP-level models, age and sex did not show consistent, independent effects. However, given the limited sample size, both negative findings and the observed rs2075507–LEDD association should be considered exploratory and interpreted with caution, pending validation in larger independent cohorts. 

The primary limitation of this study is the relatively small sample size, which may reduce the statistical power, haplotype inferences and generalizability of the findings. To validate our results, future research should include larger, more representative, and randomized cohorts. Additionally, sex distribution between control and patient groups warrants careful consideration, as imbalances may influence observed associations. In light of these limitations, our study should be considered exploratory. We intend to extend this research using larger datasets, with particular emphasis on investigating sex-specific differences among individuals with Parkinson’s disease.

## Figures and Tables

**Table 1:** Demographic and clinical characteristics of patients with Parkinson’s disease and control subjects.

Parkinson Group			Control Group	*p*-Value
Age (mean ± SD)	All	70.86 ± 7.21	66.37 ± 10.54	0.053
	Male	70.68 ± 7.87	65.86 ± 9.77	0.148
	Female	71.22 ± 5.6	66.72 ± 11.00	0.227
Gender (N)	Male	37	21	0.007
	Female	18	32	
Age at onset (mean ± SD)	All	61.62 ± 8.91	-	-
	Male	61.41 ± 9.76	-	-
	Female	62.06 ± 6.83	-	-
LEDD (mg/24 h)	All	1362.16 ± 642.13	-	-
	Male	1373.486 ± 606.057	-	-
	Female	1338.89 ± 710.00	-	-
Disease duration (years ± SD)	All	9.24 ± 5.34		
	Male	9.27 ± 4.96	-	-
	Female	9.17 ± 6.31	-	-
Hoehn and Yahr scale score (mean ± SD)	All	2.86 ± 0.82	-	-
	Male	2.87 ± 0.7	-	-
	Female	2.83 ± 1.01	-	-
UPDRS Part III score	All	31.55 ± 12.4	-	-
	Male	31.30 ± 11.94	-	-
	Female	32.06 ± 13.29	-	-
UPDRS Part IV score	All	6.31 ± 4.24	-	-
	Male	6.14 ± 4.10	-	-
	Female	6.67 ± 4.51	-	-

**Table 2:** Genotype and allele frequencies of three polymorphisms in the subjects with Parkinson’s disease and controls.

Marker	Parkinson Group			Control Group	
rs2075507 G > A	Allele	N	%	N	%
	AA	12	21.82	18	33.96
	GA	30	54.55	26	49.06
	GG	13	23.63	9	16.98
	Allele frequency—A	54	49.09	62	58.49
	Allele frequency—G	56	50.91	44	41.51
	HWE–*p*-value	1 ± 0.0001		0.597 ± 0.0005	
	Genotype distribution, PD vs. control, *p*-value	0.338 ± 0.002			
	Allele distribution, PD vs. control, *p*-value	0.178 ± 0.002			
rs4680 G > A	AA	18	32.73	10	18.87
	GA	25	45.46	35	66.04
	GG	12	21.81	8	15.09
	Allele frequency—A	61	55.45	55	51.89
	Allele frequency—G	49	44.55	51	48.11
	HWE–*p*-value	0.588 ± 0.001		0.0280 ± 0.0001	
	Genotype distribution PD vs. control, *p*-value	0.091 ± 0.001			
	Allele distribution, PD vs. control, *p*-value	0.685 ± 0.001			
rs165599 A > G	AA	23	41.82	24	45.28
	GA	27	49.09	25	47.17
	GG	5	9.09	4	7.55
	Allele frequency—A	73	66.36	73	68.87
	Allele frequency—G	37	33.64	33	31.13
	HWE–*p*-value	0.559 ± 0.001		0.334 ± 0.001	
	Genotype distribution, PD vs. control, *p*-value	0.959 ± 0.000			
	Allele distribution, PD vs. control, *p*-value	0.772 ± 0.001			

HWE = Hardy–Weinberg equilibrium analyzed using Fisher exact test. Significant *p*-values are defined as *p* ≤ 0.05 after Holm correction for multiple testing. Italicized *p*-values indicate nominal significance only at the 0.05 level.

**Table 3:** Analysis of the allele frequency distribution of the rs4680 and rs165599 polymorphisms across rs2075507 genotypes.

rs2075507				
SNP	Genotypes	GG	GA	AA
rs4680	AA	9	9	0
	GA	3	18	4
	GG	1	3	8
	2N	26	60	24
	Allele A (frequency)	21 (0.808)	36 (0.600)	4 (0.167)
	Allele G (frequency)	5 (0.192)	24 (0.400)	20 (0.833)
rs165599	AA	10	12	1
	GA	3	17	7
	GG	0	1	4
	2N	26	60	24
	Allele A (frequency)	23 (0.885)	41 (0.683)	9 (0.375)
	Allele G (frequency)	3 (0.115)	19 (0.317)	15 (0.625)
Allele frequency comparisons between rs2075507 genotypes *				
		rs2075507; AA vs. GA	rs2075507; AA vs. GG	rs2075507; GA vs. GG
rs4680	*p*-value	**0.0006**	**0.0001**	0.083
rs165599	*p*-value	**0.0002**	0.014	0.062

* Analysis performed using Fisher’s exact test. Significance level: *p* ≤ 0.05. Values in bold remain significant after Holm correction for multiple testing. Numbers in parentheses represent relative allele frequencies.

**Table 4:** Analysis of the rs4680-rs165599 haplotype frequency distribution across rs2075507 genotypes.

Haplotypes (rs4680-s165599)	rs2075507: GG	rs2075507: GA	rs2075507: AA
A-A	0.766 ± 0.083	0.581 ± 0.063	0.167 ± 0.075
G-A	0.118 ± 0.062	0.103 ± 0.04	0.208 ± 0.088
G-G	0.073 ± 0.053	0.297 ± 0.057	0.625 ± 0.098
A-G	0.042 ± 0.043	0.02 ± 0.02	-
Haplotype frequency comparisons between rs2075507 genotypes *			
	AA vs. GA	AA vs. GG	GA vs. GG
*p*-value	**0.002**	**<0.0001**	0.099

Values are mean haplotype frequencies ± standard error. * Comparisons were performed using Fisher’s exact test. Significant *p*-values (≤0.05) after Holm correction for multiple testing are shown in bold.

## Data Availability

The authors confirm that the data supporting the findings of this study are available within the article and its Supplementary Materials.

## Supplementary Materials

The following supporting information can be downloaded at: https://www.mdpi.com/article/10.3390/biomedicines14020262/s1, Table S1: Primers; Table S2: Linkage disequilibrium; Table S3: Haplotype freq. compar; Table S4: Multivar. anal. 1; Table S5: Analysis of clin. Oucomes; Table S6: Multivar. anal. 2; Table S7: Multivar. anal. 3.
